# Drop Friction on Textured Lubricant-Coated Surfaces

**DOI:** 10.1021/acsami.5c08905

**Published:** 2025-10-08

**Authors:** Xiaoyu Chen, Biruk Teka Gidreta, Tanner Gaw, Michal Remer, Dan Daniel, Xiaoguang Wang, Solomon Adera

**Affiliations:** † Energy Transport Lab, Department of Mechanical Engineering, 1259 University of Michigan, Ann Arbor, Michigan 48109, United States; ‡ Department of Chemical Engineering, University of Michigan, Ann Arbor, Michigan 48109, United States; § Institute of Aeronautics and Applied Mechanics, 49566 Warsaw University of Technology, Warsaw 00-661, Poland; ∥ Division of Physical Sciences and Engineering, King Abdullah University of Science and Technology (KAUST), Thuwal 23955, Saudi Arabia; ⊥ Department of Chemical and Biomolecular Engineering, 2647 The Ohio State University, Columbus, Ohio 43210, United States

**Keywords:** drop friction, Landau–Levich–Derjaguin (LLD) law, white-light interferometry, reflection interference contrast microscopy, lubricant-infused surface

## Abstract

Understanding drop friction on textured surfaces has implications in microfluidics and lab-on-a-chip devices. In this work, we investigated the drop friction on lubricant-coated pillars by systematically varying pillar height and density. First, we measured the friction force on a moving drop using a cantilever force sensor that has ±0.1 μN sensitivity. This measurement shows that drop friction on tall dense pillars is comparable to drop friction on short pillars, a significant result that suggests the presence of a Landau–Levich–Derjaguin (LLD) film underneath the moving drop. Second, we validated the force measurement by estimating the lubricant layer thickness by using white-light interferometry. Third, we visualized the lubricant film underneath the moving drop using reflection interference contrast microscopy. The three independent diagnostic tools and measurement techniques complement each other and reaffirm that drops oleoplane on tall dense pillars, while they graze over the pillar tops in tall sparse pillars. The critical density that forces this transition to drop friction is ≈50%. Furthermore, the experimental results show that friction on microholes and micropillars is comparable when the solid fraction is the same. The results reported in this study contradict past studies that reported the absence of an oil layer on tall pillars. Besides improving current understanding, the insights gained from this work provide design guidelines for turning drop friction on–off on demand for microfluidics applications.

## Graphical abstract

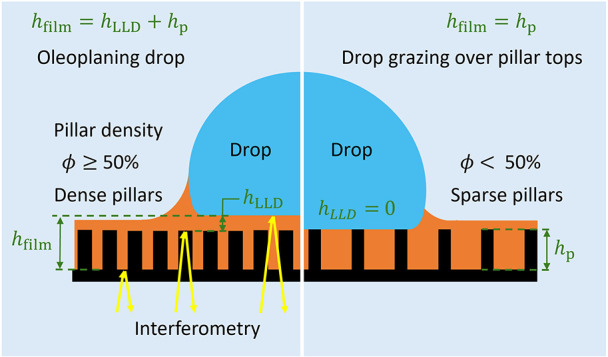

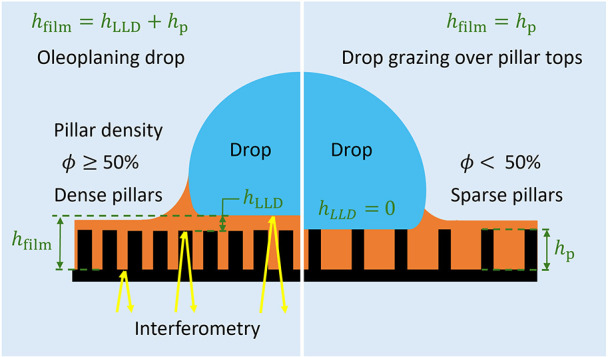

## Introduction

Naturally occurring surfaces, including various plant leaves, water strider legs, troughs on the elytra of desert beetles, and gecko feet, are superhydrophobic,^1–4^ displaying >150° apparent contact angle and ≈10–15° contact angle hysteresis.^5–8^ Some of these naturally occurring surfaces use a thin layer of oily material to coat their outer surfaces to lower the surface energy of the underlying substrate.^3,9^ This gives these surfaces the ability to effectively repel water, a variety of other fluids, and even solid particles, giving rise to an extraordinary self-cleaning behavior.^10–12^ The cutting-edge development of synthetic liquid-repellent surfaces is primarily inspired by mimicking the hierarchical roughness of lotus leaves. In literature, this effect, which has been studied extensively in the last few decades, is known as the lotus effect.^1,13–15^ Traditional lotus leaf-inspired textured water-repellent surfaces have received significant attention in past studies due to their potential to improve various engineering systems ranging from hydrodynamic drag reduction and fouling prevention to heat transfer augmentation.^16–18^

There has been significant effort toward engineering surfaces that mimic the hydrophobicity of lotus leaves while reducing contact angle hysteresis to improve drop mobility beyond the Cassie state.^19,20^ These efforts have led to recent advances in wetting science that enabled the development of nature-inspired micro-/nanostructured lubricant-coated liquid-like surfaces.^21,22^ This new class of composite material design gives rise to remarkable drop mobility by significantly reducing contact angle hysteresis.^21–24^ By providing an atomically smooth and chemically homogeneous liquid–liquid interface, the lubricant coating improves drop mobility and reduces contact line pinning.^25,26^ In fact, recent studies have reported record-low near-zero (≈1–2°) contact angle hysteresis,^27–29^ which is a proxy for minimizing solid–liquid contact and the resulting drop friction. These micro-/nanotextured lubricant-infused composite surfaces, however, still exhibit nonzero friction, the nature of which is not fully understood to date.^30–33^

Along with the extreme drop mobility that emerges as a direct outcome of lubricating the micro- and nano-surface crevices with an immiscible lubricant comes complex fluid-structure interactions and new features that are not well understood. For example, the concept of wetting ridge,^34–36^ which is the direct outcome of the unbalanced surface tension force in the vertical direction, is irrelevant in traditional air-filled superhydrophobic surfaces even though it is an important feature that plays a pivotal role in drop mobility on lubricant-coated surfaces.^37,38^ Additional features arise when analyzing drop motion on lubricant-coated surfaces due to the complex interplay between surface tension, viscosity, and the various interfacial forces at the three-phase contact line.^39–41^

Past studies have shown that the viscosity ratio between the drop and the lubricant plays a major role on the nature of drop friction on lubricant-coated surfaces.^31,42^ When the drop is more viscous than the lubricant, drop friction typically scales linearly with velocity (Stokes-type friction),^31^ whereas when the lubricant is more viscous than the drop, friction exhibits a nonlinear behavior. On such viscous lubricants, where the lubricant viscosity exceeds the drop viscosity, the viscosity-induced drop friction depends heavily on the presence or absence of an intercalated oil film underneath the drop. These studies have shown that the oil film thickness underneath a moving drop scales with the velocity to the 2/3 power, a result that follows the classical Landau–Levich–Derjaguin (LLD) scaling law.^43^ This is the same scaling law that governs the thickness of the liquid film that adheres on a flat plate that is pulled out of a viscous liquid^44–46^ or a liquid film pressed against the wall of a capillary tube by an ascending vapor bubble due to buoyancy in a gravitational field.^47^

In this paper, the LLD-governed oil film thickness due to drop motion is referred to as the critical thickness with a corresponding notation *h*_LLD_. This critical thickness, which is determined purely by the radius and kinematics of the drop motion, scales with the product of the drop radius (*R*) and capillary number (*Ca*) to the 2/3 power as *h*_LLD_ ∼ *RCa* ^2/3^. The dimensionless capillary number, which compares viscosity to surface tension, is given by *Ca* = η_o_ *U*/γ_ow_, where η_o_ is the oil viscosity, *U* is the drop velocity, and γ_wo_ is the drop-oil interfacial tension. From here on, pillars that are taller than the critical thickness (*h*_p_ > *h*_LLD_) are referred to as tall pillars, whereas pillars that are shorter than the critical thickness (*h*_p_ < *h*_LLD_) are referred to as short pillars.

For a millimeter-size water drop moving on a lubricant-coated surface, prior studies have shown that the presence or absence of an LLD-governed intercalated oil film underneath the drop is determined solely by the height of the pillars.^30,48^ When the pillar height (*h*_p_) is smaller than the critical thickness (*h*_p_ < *h*_LLD_), the drop oleoplanes on an oil layer fully separated from the underlying substrate akin to car tires on wet road.^30,48^ Moreover, these studies have shown that when the pillar height exceeds the critical thickness (*h*_p_ > *h*_LLD_), the moving drop glides over the pillar tops without forming an LLD film above the pillars.^48^ Here, we refute the latter description by providing experimental evidence.

In this work, we show that the presence of the intercalated LLD oil layer beneath a moving drop depends not only on the pillar height, as reported in prior studies, but on the pillar density. Our experimental results, which involve drop friction force and lubricant layer thickness measurements besides qualitative visualization using reflection interference contrast microscopy (RICM),^49^ show the presence of an LLD oil film on tall pillars (*h*_p_ > *h*_LLD_) when the pillar density exceeds ≈50%. From here on, this micropillar density (50%) is referred to as the critical density that triggers additional friction due to levitation of the moving drop on an oil film. We measured drop friction using a calibrated cantilever force sensor that has sub-micronewton sensitivity. Such force sensors have been used for measuring small forces on moving drops.^48,50^ The insights gained from this work improve the current state-of-the-art understanding of drop friction on textured lubricant-coated surfaces.

## Results and Discussion

A water drop on a lubricant-infused surface^21^ is accompanied by an annular wetting ridge (Figure 1a),^24,51^ the root cause of which is believed to be the imbalance of interfacial forces at the contact line. The omnipresent axisymmetric wetting ridge, which is a low-pressure region by virtue of the inward curvature of the oil–air interface, grows in size by siphoning oil from the surrounding area using capillary forces.^52–54^ Past studies have shown that the front foot (leading edge) of the wetting ridge contributes to drop friction irrespective of the pillar height due to the dynamic contact angle θ (Figure 1b) that escorts the moving drop.^30^ This dynamic contact angle has been shown to closely follow the classical Tanner’s 1/3 scaling law,^55^ which is given by θ ∼ *Ca* ^1/3^, where *Ca* is the capillary number. On the other hand, the viscosity-induced dissipation force at the rear of the wetting ridge (trailing edge) exists only in the presence of an LLD film above the pillar tops as depicted schematically in Figure 1a,b,d.^30,48^ Note the omission of Figure 1c in this description, which will be discussed in detail in subsequent paragraphs.

**Figure 1:**
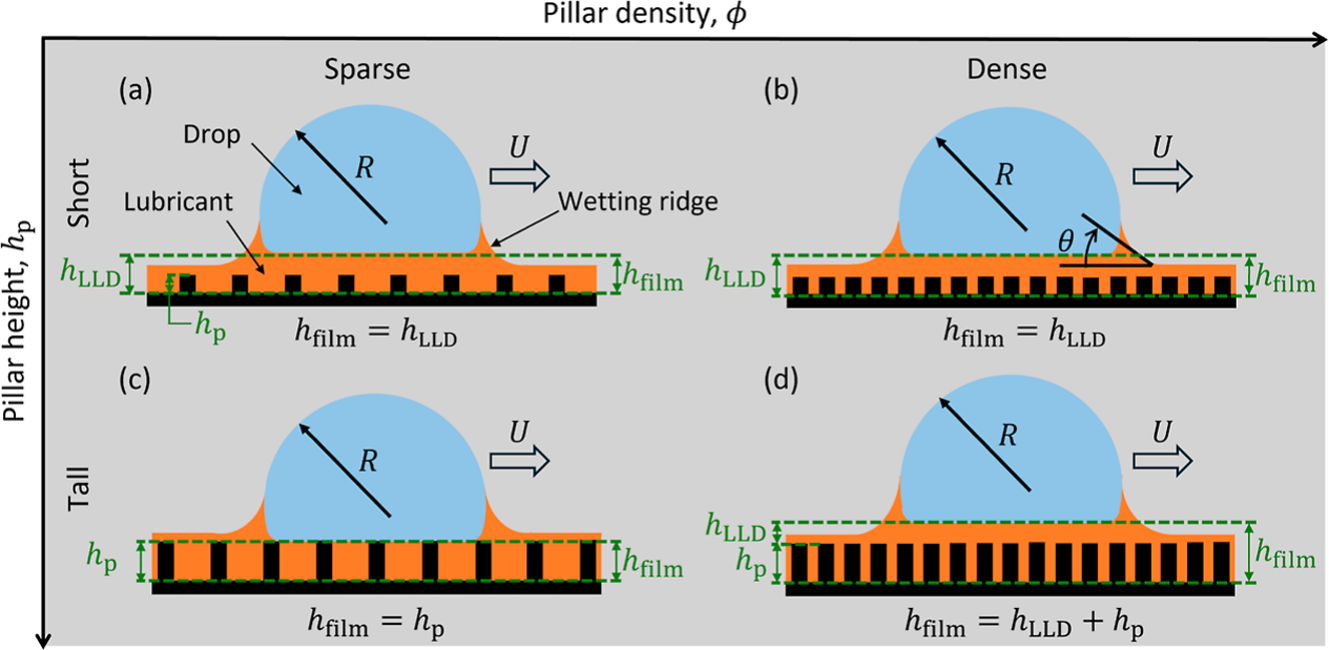
Schematic of moving drops. (a,b) Moving drops on short pillars (*h*_p_ < *h*_LLD_) oleoplane on an oil film above the pillar tops. The thickness of the oil film from the substrate base to the lubricant–drop interface obeys the LLD law and *h*_film_ = *h*_LLD_. (c) Moving drops on tall pillars (*h*_p_ > *h*_LLD_) graze over the pillar tops when the pillars are sparse (ϕ < 50%, where ϕ is pillar density). In this case, the oil film thickness is the same as the pillar height (*h*_film_ = *h*_p_). (d) Moving drops on tall (*h*_p_ > *h*_LLD_) dense (ϕ > 50%) pillars oleoplane on an oil film above the pillar tops. This oil layer thickness obeys the LLD law and the total thickness from the pillar base becomes *h*_film_ = *h*_LLD_ + *h*_p_.

For tall pillars where *h*_p_ > *h*_LLD_, it was reported that the rear foot of the wetting ridge does not contribute to drop friction due to the absence of an LLD film beneath the moving drop.^30^ In such a scenario, which is depicted schematically in Figure 1c, drop friction comes solely from the dynamic contact angle θ at the leading edge of the wetting ridge.^30^ Importantly, the moving drop on tall pillars, regardless of the pillar density, has been assumed to graze over the pillar tops without an LLD film, as shown by the schematic in Figure 1c. It is noteworthy to mention that pillar density has not been considered in past studies.^30,31,48^

This work shows that tall sparse pillars (Figure 1c) and tall dense pillars (Figure 1d) exhibit different drop friction behavior. A moving drop on tall sparse pillars grazes over the pillar tops (*h*_film_ = *h*_p_, Figure 1c). Since the LLD film thickness is less than the pillar height (*h*_LLD_ < *h*_p_), there exists no overlaying lubricant layer on top of the pillars, and the lubricant layer thickness is set by the pillar height (*h*_film_ = *h*_P_). However, a moving drop on tall dense pillars oleoplanes on an LLD-obeying intercalated oil film (*h*_film_ = *h*_p_ + *h*_LLD_, Figure 1d). Note that sparse and dense pillars are in comparison to the 50% critical pillar density. By balancing the viscous force in the front foot (leading edge) with the capillary force, friction on tall pillars (*h*_p_ > *h*_LLD_) is found to scale with ∼ϕ^2/3^ *Ca* ^2/3^, where ϕ is the solid fraction or pillar density of the micropost array. We systematically investigated the effect of pillar density by fabricating well-defined silicon micropillars and microholes using standard clean room silicon fabrication techniques. The fabrication protocol along with a scanning electron micrograph (SEM) image is provided in Supporting Information Section S1.

Experiments were conducted on well-defined lubricant-coated micropillars and microholes with varying solid fraction (0–80%), pillar height and/or hole depth, drop radius, and drop velocity to cover a wide range of capillary numbers. A typical experiment involves a millimeter-size water drop (*R* ≈ 1 mm) translating linearly at *U* ≈ 2 mm/s. In preparation for the experiments, the well-defined silicon micropillars and microholes are impregnated with silicone oil (η_o_ ≈ 20 mPa·S). A water drop (volume = 1–5 μl) is gently placed on the oil-coated surface using a micropipet. The oil–water interfacial tension measured using the pendant drop method is γ_ow_ ≈ 40 mN/m. For this scenario, the corresponding LLD film thickness becomes *h*_LLD_ ≈ $R{\left(\eta_{o}U/\gamma_{ow}\right)}^{2/3}$ ≈ 10 μm. A cantilever force sensor is used to measure the force experienced by the moving drop on the oil-coated surfaces.^32,48,56,57^ A detailed description of the force sensor calibration is shown in Supporting Information Section S2.

Drop friction measurements were conducted on tall pillars and holes with various solid fractions, as shown in Figure 2a. In agreement with past studies, the friction force measured on sparse tall pillars (red squares, ϕ ≈ 10%) align well with the semianalytical model for tall pillars (red dotted line, Figure 2a).^30,48^ The data points for tall dense pillars (blue +) and tall dense holes (green triangles), however, align well with the semianalytical model for short pillars than that for tall pillars. This result suggests the presence of a lubricant layer underneath dense pillars and holes. Drop friction is higher for short pillars than for tall pillars due to the additional source of friction from the trailing foot of the wetting ridge, which forms when there is a lubricant layer above the pillar tops.

**Figure 2:**
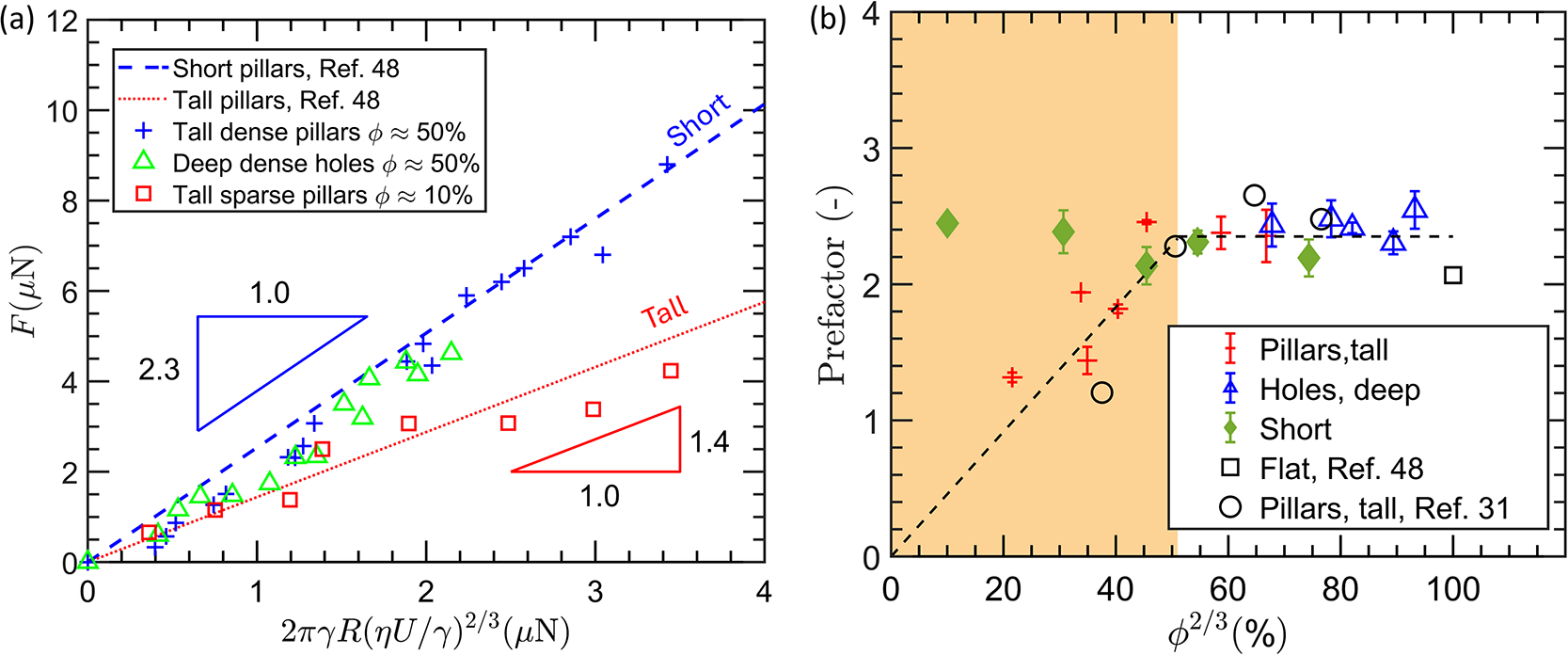
Drop friction on tall and short pillars. (a) Measured drop friction on tall dense pillars and deep holes follow the analytical model for short pillars. This indicates the presence of an LLD film beneath the moving drop on tall dense pillars. (b) Plotting a prefactor *F*/2πγ_ow_ *RCa* ^2/3^ against the pillar density shows that friction increases linearly with ϕ^2/3^ for tall sparse pillars. When the pillar density exceeds 50%, however, drop friction becomes insensitive to pillar density, suggesting the presence of an LLD oil film on pillar tops. The prefactor for tall dense pillars converges to that of the short pillars and flat silicon, suggesting the presence of an oil layer above the pillar tops.

Friction on lubricant-coated pillars depends on the radius of the drop and the capillary number following the scaling

1 $$F∼2\pi \gamma_{ow}RCa^{2/3}$$

where *R* is the radius of the drop. In the absence of an oil layer above the pillar tops, the drops are in contact with the pillar tops. As a result, the drop friction depends on the pillar density and the corresponding scaling becomes

2 $$F∼\phi^{2/3}2\pi \gamma_{ow}RCa^{2/3}$$

The terms in the equations can be rearranged to define a prefactor by using *F*/2πγ_ow_ *RCa* ^2/3^. This prefactor for a range of experiments with varying pillar density (0–80%) and pillar height (2 and 20 μm) is shown in Figure 2b. Our force measurement on tall pillars shows that when the pillar density is lower than the critical density (ϕ < 50%), the prefactor increases linearly with ϕ^2/3^ as expected from eq 2. This increase in the prefactor levels off and becomes nearly constant when the pillar density exceeds the critical solid fraction of 50%. Note that the prefactor for short pillars is constant regardless of the pillar density as shown by the green data points in Figure 2b. This measurement also agrees well with the force scaling given in eq 1. The convergence of the prefactor of the tall dense pillars toward the short pillars beyond the critical pillar density suggests the presence of an oil layer above the pillar tops when the pillars are dense. These measurements agree with force measurements from prior studies including the ones that use flat silicon surfaces, confirming the validity of our measurement and data interpretation.^48^

To validate our force measurement, we carried out an independent experiment where the lubricant film thickness underneath a moving drop was measured using white-light (200–850 nm) interferometry on tall sparse and dense pillars (Figure 3a,b). For this experiment, micropillars of varying dimensions were fabricated on a transparent substrate by using conventional soft-lithography. The white light that goes through the substrate is reflected back from the substrate–lubricant and drop–lubricant interfaces as shown schematically in Figure 3a. Reflection from the substrate–air interface is suppressed by using immersion oil, which matches the refractive index of the substrate. Due to the surface topography, two distinct reflections were captured: one from the pillar tops and another from the pillar base (see Supporting Information Section S3). This strategy enabled us to measure the oil film thickness from the bottom of the pillar (*h*_film_) and the top of the pillar (*h*_1_) with up to 0.1 μm accuracy as shown schematically in Figure 3a. The difference between *h*_film_ and *h*_1_ matched the pillar height *h*_p_ measured by using SEM, confirming the validity of the measurement. Importantly, for tall dense pillars, the thickness of the lubricant film above the pillar tops, *h*_1_, matches the kinematically determined LLD film thickness as shown in Figure 3a. However, for tall sparse pillars, *h*_1_ ≈ 0, and the film thickness, *h*_film_, is set by the pillar height (*h*_film_ = *h*_p_) as shown in Figure 3b. This result agrees well with our force measurements.

**Figure 3:**
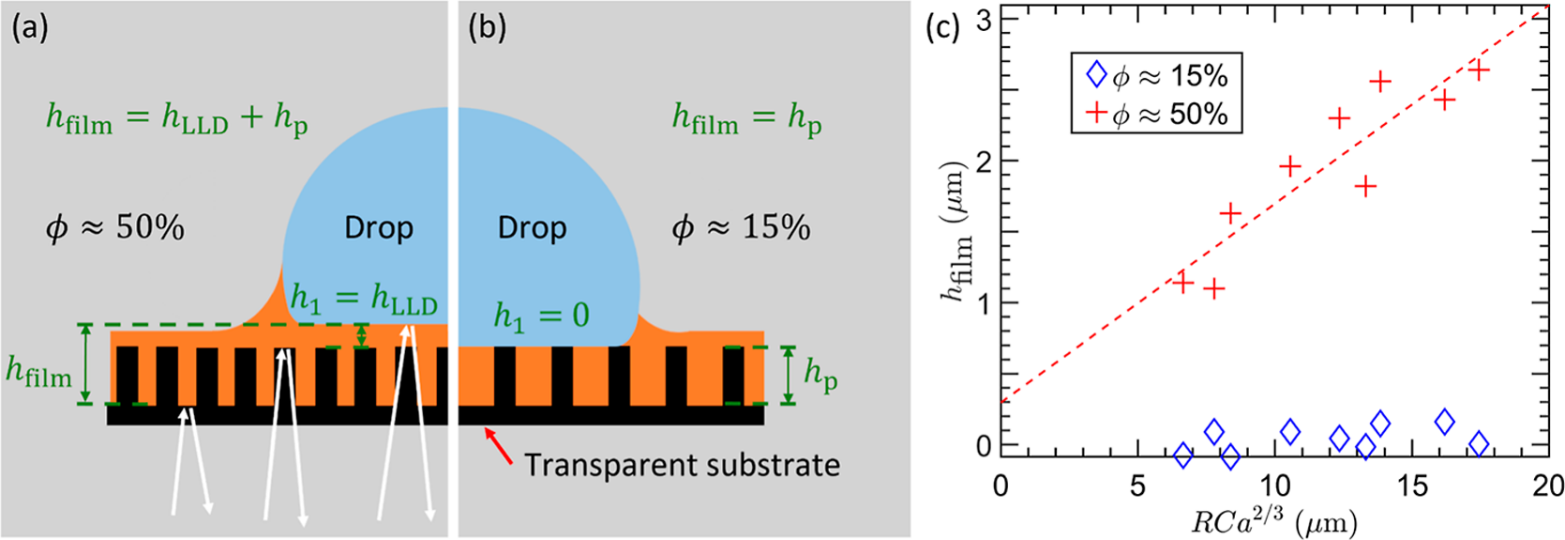
Lubricant layer thickness. (a) Schematic of the white-light interferometry used to measure the lubricant layer thickness. The reflection from the different interfaces was analyzed to deduce the oil thicknesses *h*_1_ and *h*_film_. Tall dense pillars form an LLD film above the pillar tops (*h*_film_ = *h*_LLD_ + *h*_p_). (b) Tall sparse pillars graze over the pillar tops (*h*_film_ = *h*_p_) with *h*_1_ ≈ 0. (c) Film thickness measurements on tall sparse and dense pillars. On dense pillars, a lubricant layer of thickness *h*_1_ ≈ 1–3 μm forms above the pillar tops. Importantly, the film thickness obeys the LLD law (shown in the red dashed line) and scales with 2/3 power of the capillary number as *RCa* ^2/3^. On the sparse pillars, however, the lubricant layer thickness was *h*_1_ ≈ 0 regardless of the velocity of the drop.

For a drop oleoplaning over a layer of lubricant, the film thickness increases with velocity to the 2/3 power following the LLD law using the scaling

3 $$h_{LLD}∼RCa^{2/3}$$

On the tall sparse pillars (ϕ ≈ 15%), the film thickness *h*_1_ ≈ 0 and has no dependency on the velocity of the drop as shown by the blue data points in Figure 3c. However, for tall dense pillars (ϕ ≈ 50%), *h*_1_ is nonzero and increases with velocity in accordance with the LLD law as shown by the red data points in Figure 3c. These results further confirm the presence of an LLD film on pillar tops when the pillar density exceeds 50%.

Using a transparent substrate, we visualized the lubricant film intercalated between the drop and the underlying substrate using reflection interference contrast microscopy (RICM).^60,61^ This visualization technique is discussed in detail in Supporting Information Section S4. Briefly, a monochromatic light with a wavelength λ = 470 nm was shone from beneath the moving drop, and the reflected light was captured through the pinhole of a confocal microscope with a 10× objective lens. In the presence of a lubricant film beneath the drop, the reflected light beams interfere constructively or destructively to form bright and dark fringes. The RICM images of a moving drop on varying height and density of pillars are shown in Figure 4 and in Supporting Information Movie S1. For short pillars, the interference fringes were visible irrespective of the pillar density, as shown in Figure 4a,b. For tall pillars, however, the interference fringes were present for dense pillars (Figure 4c) and absent for sparse pillars (Figure 4d). This is additional and independent confirmation that the drops oleoplane on a lubricant film when the pillars are dense. The visualization experiments in Figure 4 complement our force measurement and agree with our interpretation of the data.

**Figure 4:**
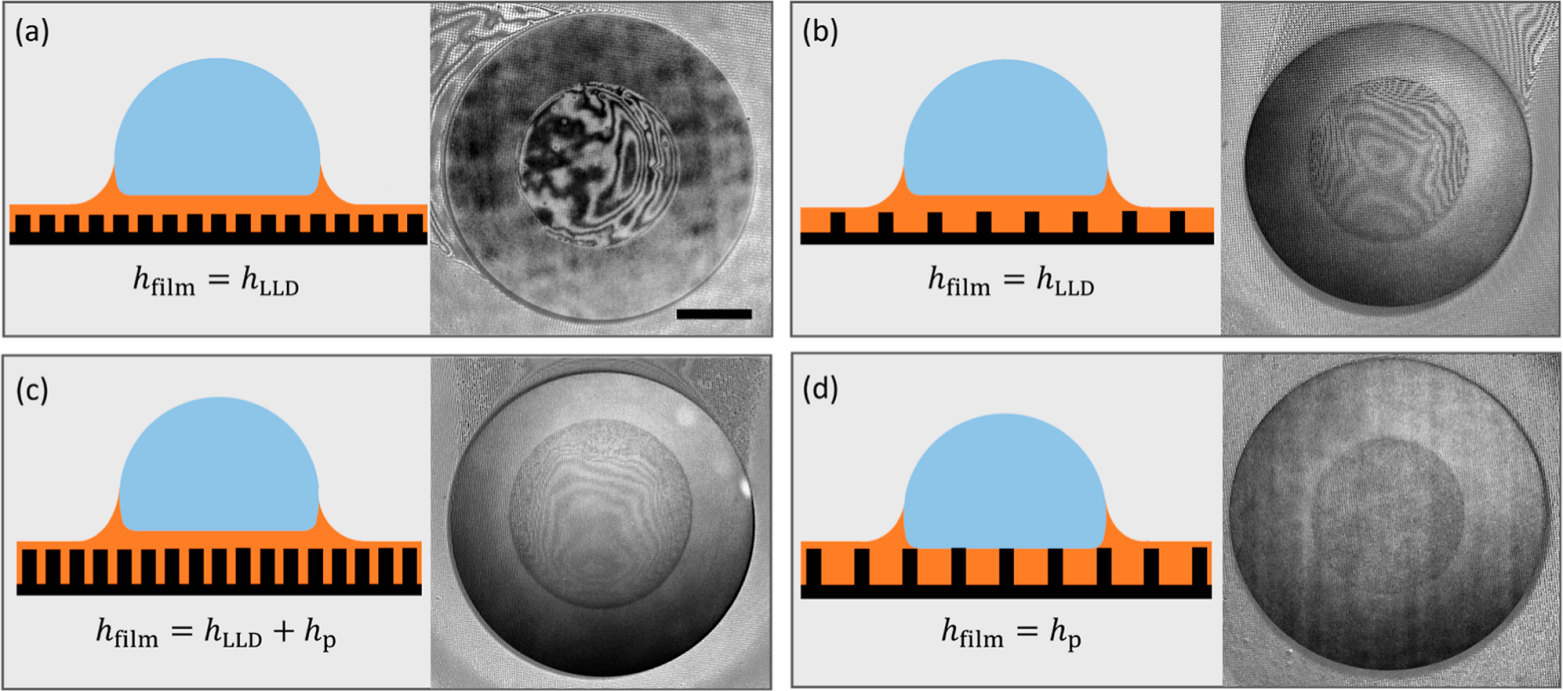
Visualization of the intercalated lubricant film. The lubricant film underneath an oleoplaning drop was visualized using reflection interference contrast microscopy (RICM). The schematics on the left side of each panel show the side view of the moving drops (not to scale). A 1 μl water drop moving at 10 mm/s on (a) short dense pillars, (b) short sparse pillars, (c) tall dense pillars, and (d) tall sparse pillars. For this visualization experiment, the heights of the pillars were 2 and 20 μm while the densities were 20% and 70%. The intercalated oil film sandwiched between the drop and the underlying substrate is visible in the RICM images in (a), (b), and (c) while it is absent in (d). Tall sparse pillars do not allow the LLD film to form on top of the pillars and the oil film thickness becomes the same as the pillar height, a result that agrees with our force measurement. The 3 mm scale bar in (a) applies to all figures.

Additionally, we measured the friction force on moving drops on micropillars and microholes.^62^ The measurements show that the drop friction is invariant with the underlying surface geometry. In our measurement, the drop friction on micropillars and microholes with the same solid fraction was comparable. This observation agrees well with our previous work that shows insensitivity of drop impact dynamics on the geometry and arrangement of silicon micropillars.^63^ This is shown in Figure 5 for dense micropillar and microhole array structures with a solid fraction of ≈50%. The experiments also show that friction on both micropillars and microholes follows the scaling law proposed in the literature.^48^ In these experiments, the drop size, velocity, and lubricant viscosity were varied to cover a wide range of capillary numbers.

**Figure 5:**
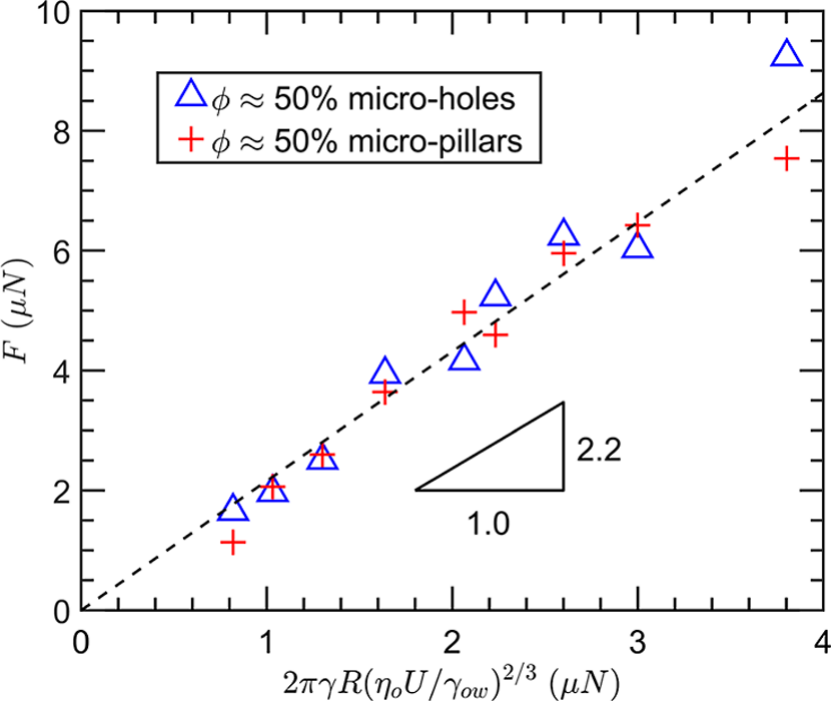
Drop friction on micropillars and microholes. Drop friction on micropillars and microholes of the same solid fraction is comparable. Friction depends only on drop radius and velocity following the scaling $F∼2\pi \gamma_{ow}R{\left(\eta_{o}U/\gamma_{ow}\right)}^{2/3}$ .

## Conclusion

In this work, we studied drop friction on lubricant-coated micropillars by systematically varying the pillar height and pillar density. Our measurements show the presence of a Landau–Levich–Derjaguin (LLD) lubricant layer beneath a moving drop on tall dense pillars, a result that contradicts prior studies. Unlike our report, past studies show the absence of an oil film underneath a moving drop on tall pillars, regardless of pillar density. Our experiments, which show otherwise, are confirmed by directly measuring the friction force and the lubricant layer thickness on moving drops besides visualizing the lubricant layer sandwiched between the drop and the underlying substrate using reflection interference contrast microscopy (RICM). Force measurements show that drop friction increases with pillar density for tall sparse pillars, while it remains nearly identical for tall dense pillars. Importantly, the drop friction on tall dense pillars is comparable to that on short pillars, a result that suggests the presence of an intercalated oil layer underneath the moving drop. This result was further confirmed by measuring the lubricant layer thickness using white-light interferometry. On tall sparse pillars, we measured consistent lubricant film thickness that matches the pillar height *h*_film_ ≈ *h*_p_, regardless of the drop velocity, whereas on tall dense pillars, the lubricant thickness exceeds the pillar height *h*_film_ > *h*_p_. Importantly, the overlayer lubricant film thickness above the tall dense pillars increases with the velocity to 2/3 power, a result that is reminiscent of the classical LLD law. We further confirmed the force and thickness measurements using reflection interference contrast microscopy (RICM). The RICM visualization confirmed the presence of an intercalated lubricant film atop tall dense pillars, even though the pillar height is greater than the kinematically determined (based on drop radius and drop velocity) LLD film thickness. Moreover, force measurements show that the drop friction on micropillars and microholes of the same solid fraction are comparable, a major result that underscores the insensitivity of drop friction on the underlying microstructure geometry. Besides improving the current understanding in the field, the new knowledge and insights gained from this work can be used as guidelines for the rational design and fabrication of engineered surfaces that can manipulate drop-surface interaction and the resulting friction force effectively.

## Materials and Methods

### Sample Preparation

Well-defined cylindrical silicon micropillar arrays arranged in a square pattern were fabricated using standard contact photolithography and deep reactive-ion etching (DRIE).^64,65^ Following fabrication, the test samples were coated with hydrophobic nanocolloids (Glaco Mirror Coat Zero, SOFT99USA) to increase their water repellency. To cover the samples uniformly with a hydrophobic coating, the Glaco solution was first diluted by using methanol. Before coating, the samples were thoroughly cleaned using acetone, methanol, ethanol, 2-propanol, and deionized water. After wet cleaning, the samples were rinsed thoroughly using deionized water and dried using compressed nitrogen gas. This was followed by a 15 min plasma treatment (PDC-001-HP, Harrick Plasma) in a nitrogen environment. The clean and plasma-treated samples were then immersed in the solution, after which they were pulled out vertically at a controlled speed. Finally, the samples were placed in a forced air convection oven (1350 FM, VWR) that was maintained at 70 °C for 30 min. Alongside the micropillars, microholes were fabricated following the same protocol. Microholes allowed us to span a wider range of solid fractions. Note that the theoretical maximum solid fraction that can be achieved via the standard photolithography techniques for cylindrical pillars is π/4 ≈ 80%. Practical limitations on photoresist patterning and development, photolithography, and DRIE, however, do not support 80% pillar density. Moreover, microholes enabled us to experimentally characterize and compare drop friction on micropillars and microholes. The sample fabrication protocol, including scanning electron microscope (SEM) images, is provided in Supporting Information Section S1 and Figure S1.

### Force Measurement

To measure the friction force, we built a custom cantilever force sensor from an acrylic tube with inner and outer diameters of 0.210 and 0.415 mm, respectively. The sensor was first calibrated by placing a known volume of water drops at its tip and capturing deflection due to the drop weight Δ*x* using a high-resolution camera. The constant *k* is determined by examining the slope of values obtained from measurements of deflections due to various drop weights. The detailed description of the calibration procedure is provided in Supporting Information Section S2, Figures S2 and S3.

### Lubricant Thickness Measurement

In this study, we used white-light interferometry to measure the lubricant layer thickness beneath the moving drop. Briefly, white light is shone from a probe (RP21, ThorLabs), coupled to a spectrometer (FLAME-S-VIS-NIR, Ocean Insight) and broadband halogen lamp (HL-2000-LL, Ocean Insight), underneath the transparent substrate. The spectrometer is then integrated for 10 ms to measure the reflected QTH lamp spectrum. To obtain a reference spectrum, a bare sample prior to lubrication is measured in an identical fashion. The details of the white light interferometry measurements, including the experimental setup, are provided in Supporting Information Section S3 and Figure S4.

### Reflection Interference Contrast Microscopy (RICM)

To visualize the lubricant layer under the moving drop, a monochromatic light (Thorlabs) of a wavelength λ = 470 nm is used to scan the surface and the reflected light is captured through the pinhole of a confocal microscope. The objective lens (Mitutoyo) used for this visualization has 10× magnification and a 0.14 numerical aperture. The detailed description of the experimental procedure, including the experimental setup, is provided in Supporting Information Section S4 and Figure S5.

## Supplementary Material





## Notes

Details of sample fabrication, force sensor calibration, white-light interferometry, and reflection interference contrast microscopy (RICM) (PDF)
Lubricant layer visualization using reflection interference contrast microscopy (RICM) (MP4)
