# Supplementary material for: Drop Friction on Textured Lubricant-Coated Surfaces
Source: ACS Appl Mater Interfaces. 2025 Oct 8;17(43):59856–63. doi: 10.1021/acsami.5c08905 (PMC12581128; doi:10.1021/acsami.5c08905)
Supplement: Supplementary file 1 [file am5c08905_si_001.pdf]

Supplementary Information

Supplementary Information for

Drop friction on textured lubricant-coated surfaces

Xiaoyu Chen[1], Biruk Teka Gidreta[1], Tanner Gaw[2], Michal Remer[1,3], Dan Daniel,[4] Xiaoguang Wang[5], Solomon Adera[1*]

[1]Energy Transport Lab, Department of Mechanical Engineering, University of Michigan, Ann Arbor, Michigan, USA

[2]Department of Chemical Engineering, University of Michigan, Ann Arbor, Michigan, USA

[3]Institute of Aeronautics and Applied Mechanics, Warsaw University of Technology, Warsaw, Poland

[4]Division of Physical Sciences and Engineering, King Abdullah University of Science and Technology (KAUST), Thuwal, Saudi Arabia

[5]Department of Chemical and Biomolecular Engineering, The Ohio State University, Columbus, Ohio, USA

*Corresponding author: Solomon Adera
Email: sadera@umich.edu

Supplementary movies

Movie 1. Reflection interference contrast microscopy (RICM). This movie shows a 2 μl water drop oleoplaning over a textured lubricant-coated surface. As the droplet starts to oleoplane, the interference fringes (Newton rings) due to light interference become visible. The movie was recorded at 30 frames-per-second (fps) using a transparent substrate fabricated using the conventional soft lithography.

S1. Sample fabrication

In this study, we first fabricated silicon microstructures using standard contact photolithography and deep reactive ion etching. The microstructures are well-defined cylindrical micro-holes (Figure S1a) and micro-pillars (Figure S1b) arranged in a square pattern. To obtain hydrophobic surfaces, the samples were then coated with nanometric colloidal particles (Glaco Mirror Coat, Soft 99), as shown in Figure S1c. Two additional steps are taken on the bare silicon samples to fabricate transparent samples. First, a PDMS counter-mold was fabricated by transferring the texture from the silicon substrate. To achieve this first, the silicon microstructure was hydrophobized by vapor deposition of silane (trichloro (1H,1H,2H,2H-perfluorooctyl) silane, Santa Cruz Biotechnology) for an easy release of the sample. Following silanization, the sample was plasma cleaned (PDC-001-HP, Harrick Plasma) in a nitrogen environment for 15 minutes. A polydimethylsiloxane PDMS (SYLGARD 184 Silicone Elastomer) base was mixed thoroughly with a curing agent at a 10:1 ratio, and the mixture was degassed for 30 minutes. The degassed mixture was cast on the silanized sample and then cured in a convection oven (1350 FM, VWR) at 70 °C overnight. The second step was to transfer the textures on the PDMS counter mold onto a glass slide. For this process, a glass slide was thoroughly cleaned using acetone, methanol, ethanol, isopropyl alcohol, and deionized water, followed by a 15-minute plasma treatment (PDC-001-HP, Harrick Plasma) in a nitrogen environment. Then, a transparent Norland optical adhesive (NOA61) was spread over the glass slide and cast over the PDMS counter-mold, which was then cured overnight in an oven maintained at 70 °C. Finally, the transparent samples were coated with Glaco to render the samples hydrophobic. The microstructures used in this study had pillar densities ranging from 15% to 70%. Pillar density refers to the fraction of the surface area of a microstructure that is solid. i.e. the pillar density for micro-pillars is given as $\pi d^2/w^2$, while that for micro-holes is given as 1- $\pi d^2/w^2$ where d and w are the pillar/hole diameter and the center-to-center spacing, respectively.

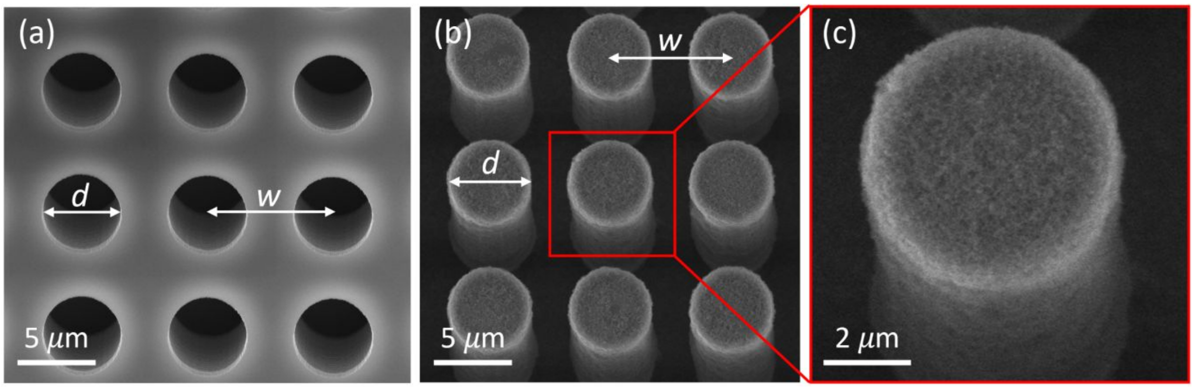

Figure S1: Surface characterization. Scanning electron microscopy (SEM) images of (a) micro-holes, (b) micro-pillars, and (c) a micro-pillar coated with nanoparticles. For RICM experiments, the bare microstructures were transferred onto glass slides and chemically treated with hydrophobic nanometer-scale colloids to obtain transparent samples.

S2. Cantilever force sensor

To measure friction force, we used a custom-built force sensor. The sensor is an acrylic capillary tube with inner and outer diameters of 0.210 mm and 0.415 mm, respectively, with a length of 11 cm. To determine the force constant k of the force sensor, we calibrated it by placing a known volume of water droplets, ranging from 0.6 to 2.5 μl, at the tip of the capillary tube as shown in Figure S2a. The deflection Δx was captured due to the droplet weight using a high-resolution camera. The constant $k$is determined by examining the slope of values obtained from measurements of deflections due to various droplet sizes. The resultant spring constant equals k = 4.2 mN/m as shown in Figure S2b. In our experiments, we were able to measure deflections as small as 0.02 mm, which allows us to measure force as small as 100 nN.

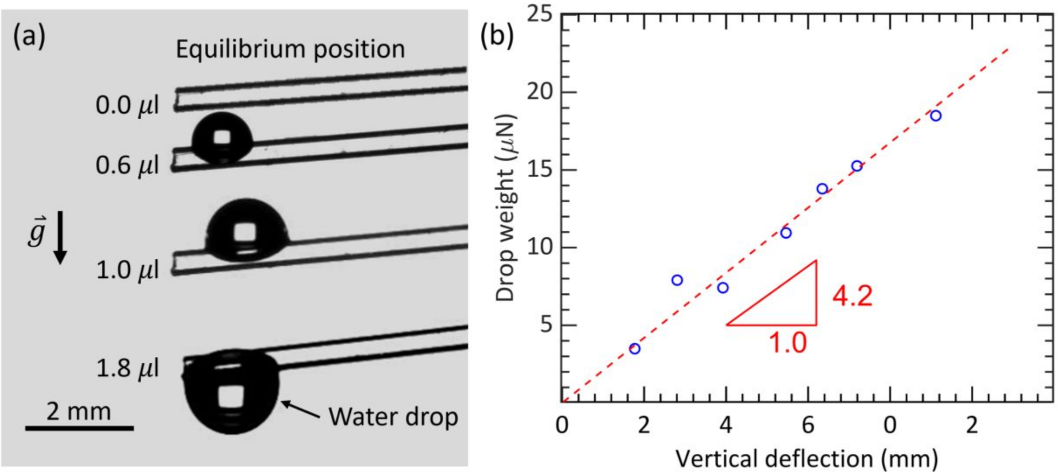

Figure S2: Calibration of the cantilever force sensor. The force sensor is modeled as a cantilever beam and assumes its deflection at the tip is linearly proportional to the applied force. (a) The force sensor is calibrated by placing a water droplet of known volume at the tip of the capillary tube. (b) By detecting the deflection using a high-resolution camera, we relate the applied force to the vertical deflection and determine the force constant. The measured force constant is k = 4.2 mN/m.

After calibrating the cantilever force sensor, we measured the friction force by placing the substrate on a linear stage moving at a known constant velocity. The experimental setup and a schematic showing the deflection of the cantilever force sensor are shown in Figure S3a, b. The droplet, placed on the top of the substrate, was kept stationary by the capillary tube, whose deflection Δx, was used to calculate the friction force F, as shown in Figure S3c.

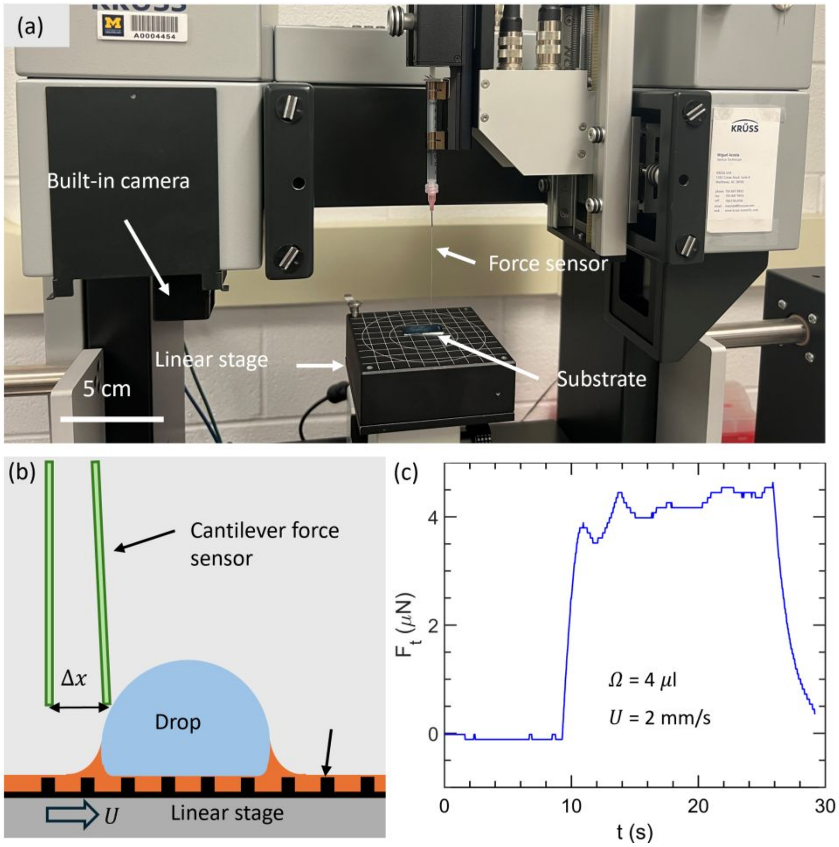

Figure S3: Force measurements. (a) The experimental setup for measuring the friction force includes a camera, linear stage, and force sensor. (b) Schematic of the experimental setup shows the deflection of the cantilever force sensor. The force sensor is an acrylic capillary tube and can be modeled as a cantilever beam. During the experiments, the surface is placed on the linear stage, moving at a constant speed, and the water droplet is kept stationary by the cantilever sensor. (c) The force on the droplet is a function of time. The linear stage starts at around 10 s. The dissipative force is measured by averaging the force for 20 s, up to 90 s, depending on the stage velocity.

S3. White light interferometry

We used white light interferometry to measure the lubricant layer film underneath the moving droplet. The schematic of the experimental setup is shown in Figure S4a. We shone a white light from a probe (RP21, ThorLabs), coupled to a pocket spectrometer (FLAME-S-VIS-NIR, Ocean Insight) and broadband quartz-tungsten halogen lamp (HL-2000-LL, Ocean Insight), placed ≈1 mm beneath the surface. The surface was placed on a stand attached to a linear stage, which moved at a constant velocity of 0.5 – 5 mm/s, while the droplet was kept in place by a capillary tube.

The spectrometer was integrated for 10 ms to measure the reflected Quartz Tungsten Halogen (QTH) lamp spectrum. An identical scan with the lamp light turned off was taken to obtain background measurements. A diffuse reflection standard (WS-1, Ocean Insight) was measured in a similar fashion to produce a reference spectrum. The background noise is subtracted from the measured intensities of the sample and reference probe, and the reflection coefficient can be calculated as

$$R(\lambda) = \frac{I(\lambda) - B(\lambda)}{I_{ref}(\lambda) - B_{ref}(\lambda)}, \quad (1)$$

where I and B are the measured intensity and the background measurement, respectively. The subscript refers to the measurement of the reference sample.
The reflection coefficient can be approximated as

$$R(\lambda) \approx r_{os}^2 + 2r_{ao}r_{os}\cos\left(\frac{4\pi n_o b}{\lambda}\right) \approx c_1 + c_2\cos(2kn_o d)\,, \tag{2}$$

Where r_{ao} and r_{os} are the Fresnel amplitude reflection coefficients of air-oil and oil-sample interfaces, respectively. n_o, b, and k are the refractive index of the oil film, the oil film thickness, and the vacuum wave number. The measured reflection spectrum is re-sampled into k-space to calculate $R(k)$. After a detrending operation is performed, the discrete Fourier transform of the filtered $R(k)$ is calculated, and a Cauchy distribution is fitted to the spectrum's peak. The location of the peak corresponds to the mean value $2n_o b$, from which the mean value of b is calculated. Our measurements on the microstructured surfaces show two peaks, b_1 and b_2, each corresponding to the thickness from the top and bottom of the microstructures, as shown in Figure S4b.

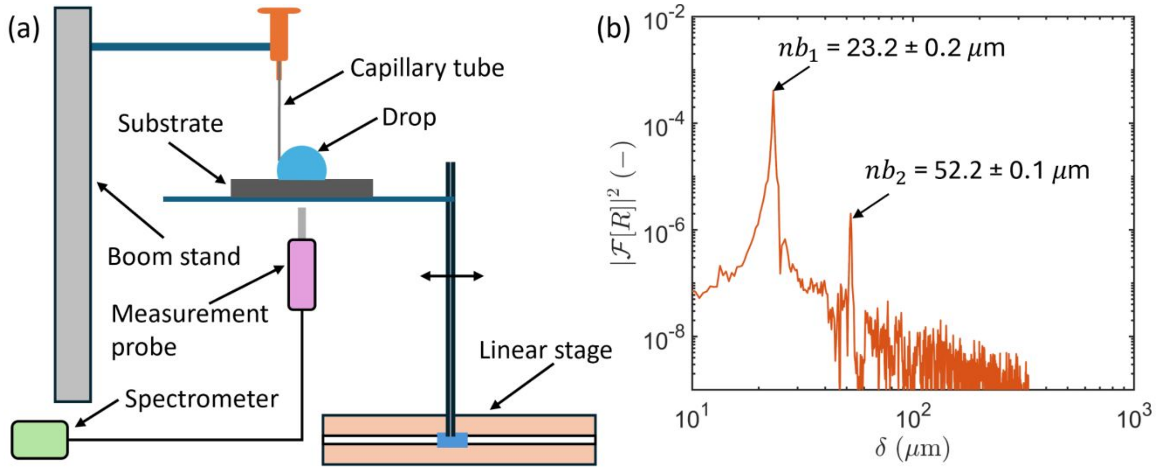

Figure S4: White light interferometry. (a) Schematic of the experimental setup for white light interferometry showing the probe coupled with a spectrometer, a lamp, and a linear stage to which a capillary tube is attached to move the droplet. (b) Fourier-amplitude spectrum of $R(k)$ showing two distinct peaks correspond to the film thicknesses measured from the top and bottom of the micro-pillars. The value of refractive index n used for estimating the intercalated lubricant film thickness is 1.41 for silicone oil.

S4. Reflection interference contrast microscopy (RICM)

Reflection interference contrast microscopy (RICM) can be used to visualize the lubricant film intercalated between the droplet and the solid substrate. Figure S5a shows the experimental setup for RICM. A monochromatic light (Thorlabs) of wavelength λ = 470 nm is used to raster scan the surface, and the reflected light is captured through the pinhole of a confocal microscope. The objective lens (Mitutoyo) used for this visualization has 10× magnification and a 0.14 numerical aperture.

In the presence of a thin film, the light reflected from the bottom (lubricant-substrate) interface and the top (lubricant-droplet) interface interfere with each other constructively or destructively to form bright and dark fringes, respectively. By detecting and analyzing these variations in brightness or contrast in the interference pattern, RICM can provide information about the thickness and distribution of the lubricant film. Additionally, changes in the interference pattern over time can reveal dynamic processes such as the spreading or movement of the droplet. In this study, RICM is used merely to determine the presence of an intercalated lubricant film; the film thickness is measured using white light interferometry, as discussed in section S2. As can be seen in Figure S5b and Supplementary Movie 1, when a droplet moves on top of lubricant-coated short pillars, an intercalated lubricant film is present (LLD film). On tall pillars, when the density is low, the droplet makes contact with the micro-pillars, and no stable lubricant film sandwiched between the droplet and the pillars exists. However, when the tall pillars are dense, we observe that an intercalated lubricant film exists on top of the pillars even though the height of the pillars is larger than the expected LLD film. This confirms that the high density of the micro-pillars prevents the lubricant from draining away between the pillars and that the surface behaves like a flat surface.

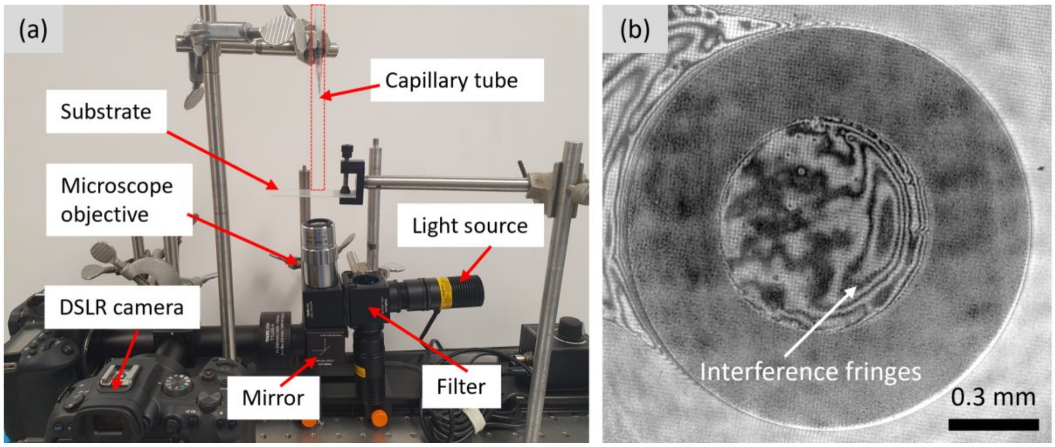

Figure S5: Reflection interference contrast microscopy (RICM). (a) The experimental setup of RICM showing a monochromatic light source (λ = 470 nm), a turning mirror (45°), a dichroic filter, and a linear stage to move the substrate while the droplet is kept stationary by a capillary tube. (b) RICM image of an oleoplaning drop shows Newton rings.
